# Safety and Functional Outcomes Following Total Hip Arthroplasty Using the Latitud™ Total Hip Replacement System in Patients With Hip Fracture

**DOI:** 10.7759/cureus.84104

**Published:** 2025-05-14

**Authors:** Ponnanna Machaiah, Supreet Bajwa, Ravi Teja Rudraraju, Kunal Aneja, Nitesh Tandiya, Shakir Kapadia, Narendra Parmar, Ashish Singh, Yogesh Chaudhari, Rashid Hasan, Vinod Arora, Shubh Mehrotra, Shailendra Patil, Ashok kumar Thakkar, Udita Chandra

**Affiliations:** 1 Orthopaedics, Ramaiah Medical College and Hospital, Bangalore, IND; 2 Orthopaedics, Wockhardt Hospital, Mumbai Central, Mumbai, IND; 3 Orthopaedics, Apollo Hospitals, Hyderabad, IND; 4 Orthopaedics, Max Super Speciality Hospital, New Delhi, IND; 5 Orthopaedics and Rehabilitation, Naveda Healthcare Centre, New Delhi, IND; 6 Orthopaedics, Barod Hospital, Indore, IND; 7 Orthopaedics, Saifee Hospital, Mumbai, IND; 8 Orthopaedics, Yashfeen Hospital, Navsari, IND; 9 Orthopaedics, Anup Institute of Orthopaedics and Rehabilitation, Patna, IND; 10 Orthopaedics, Ashoka Medicover Hospital, Nashik, IND; 11 Orthopaedics, Wockhardt Hospital, Nashik, IND; 12 Orthopaedics, Bombay Hospital, Indore, IND; 13 Orthopaedics, Medi-Square Hospital and Research Centre, Indore, IND; 14 Orthopaedics, Lovee Shubh Clinic Pvt. Ltd., Lucknow, IND; 15 Orthopaedics, Currae Hospital, Thane, IND; 16 Clinical Research and Medical Writing, Meril Life Sciences Pvt. Ltd., Vapi, IND

**Keywords:** harris hip score, hip fracture, implant survivorship, latitud total hip replacement system, oxford hip score, revision rates, total hip arthroplasty

## Abstract

Background

Hip fractures, particularly among the aging population, present a significant burden due to their association with morbidity, mortality, and long-term disability. Total hip arthroplasty (THA) is increasingly being adopted for fracture management in active patients, but implant performance data in real-world Indian settings remain sparse. This study assesses the early clinical efficacy, functional outcomes, and implant survivorship of the Latitud™ total hip replacement system (HRS) (Meril Healthcare Pvt. Ltd., Vapi, India), a novel prosthesis designed and manufactured in India, in patients undergoing THA for hip fractures.

Methods

This post hoc analysis derives from a prospective, multi-center, post-marketing surveillance study conducted across 11 tertiary care centers in India. A total of 44 adult patients presenting with various fracture patterns, including femoral neck, femoral head, intertrochanteric, sub-capital, and head-neck fractures, were evaluated following THA using the Latitud™ HRS. The primary objectives of the study were implant survivorship and adverse events over a two-year follow-up period, whereas the secondary objective of the study was to assess functional outcomes using the Harris hip score (HHS) and Oxford hip score (OHS) at six weeks, six months, one year, and two years postoperatively. Paired t-tests or Wilcoxon signed-rank tests were used for statistical comparisons, with p < 0.05 considered significant.

Results

A total of 44 patients were identified with the following fracture types: femoral neck (n = 18), femoral head (n = 8), head-neck (n = 2), and hip fractures (n = 12), with one case each of intertrochanteric, subcapital, intracapsular neck of femur (ICNF), and neck of femur fractures due to fall at home. Functional outcomes significantly improved, with the mean HHS increasing from 9.19 ± 9.52 preoperatively to 89.61 ± 3.57 (p < 0.0001), and the OHS from 4.89 ± 4.41 to 44.89 ± 3.77 (p < 0.0001). No serious adverse events were observed apart from the isolated revision case. At two years, implant survivorship was 97.7%, with a single revision due to polyethylene liner fracture. No other major complications, including infections, dislocations, or periprosthetic fractures, were reported.

Conclusion

The Latitud™ HRS demonstrated excellent short-term survivorship and marked functional improvement in patients with hip fractures, with minimal complications. These findings support its safety and effectiveness in the Indian clinical context and suggest its potential as a reliable implant for fracture-related THA. Further large-scale, long-term comparative studies are warranted to confirm these outcomes.

## Introduction

Hip fractures pose a significant challenge in orthopedic management, especially among older adults. These fractures typically result from low-impact trauma, such as falling from a standing height or lower, and are often indicative of underlying osteoporosis [1,2]. With the global increase in life expectancy, the incidence of hip fractures is anticipated to surge, highlighting a major public health concern [2]. Epidemiological studies predict a dramatic rise in hip fractures over the next few decades, with estimates suggesting up to six million cases annually by 2050, half of which are expected to occur in Asia due to the region's rapidly ageing population [2]. Moreover, hip fractures are associated with increased comorbidity and mortality rates [3]. Post-fracture mortality within one year ranges from 18% to 33%, with a fivefold to eightfold increased risk of death in the first three months compared to individuals without fractures. This elevated risk persists for up to a decade [3].

Historically, hip fractures, particularly those involving the femoral neck, have been treated with internal fixation and hemiarthroplasty. While internal fixation is less invasive, it carries risks such as non-union, avascular necrosis, and the need for reoperation [4]. These limitations have generated interest in total hip arthroplasty (THA) as a more promising treatment option. THA is widely recognized as an effective method for achieving long-term pain relief and restoring function in patients with hip pathology [5]. It is particularly recommended for patients with fractures of the femoral neck, intertrochanteric region, or acetabulum [5].

Originally designed for managing hip osteoarthritis, THA entails replacing both the femoral head and acetabulum with prosthetic components [6]. This comprehensive joint replacement technique has demonstrated superior functional outcomes. It offers the potential for enhanced pain relief, improved mobility, and a reduced likelihood of requiring reoperation [6].

Several surgical approaches for THA exist, each with its own set of advantages and drawbacks. The posterior approach is the most frequently used, providing excellent visualization of both the acetabulum and femur, and is adaptable for more extensive procedures [7]. When accompanied by proper soft tissue repair, the posterior approach does not result in a higher dislocation rate compared to other techniques [7]. However, implementing THA for hip fractures presents certain challenges. The procedure is more invasive and carries an increased risk of perioperative complications, such as infection, dislocation, and aseptic loosening [8]. Additionally, extended surgical duration and the need for intensive rehabilitation introduce further risks, particularly for older, frail patients with multiple comorbidities [2].

Selecting the appropriate implants for THA in the context of hip fractures involves complex decision-making. With increased demands for more personalized implants with varied anatomical needs, developing those with better wear characteristics remains challenging [7]. Given that instability is a major complication of THA for fracture treatment, careful consideration must be given to implant selection [9].

Critical factors influencing implant choice include the patient's age, activity level, bone quality, and existing comorbidities [10]. The surgeon's experience and familiarity with specific implant systems are also crucial considerations [10]. The primary goal is to establish a stable, well-functioning hip joint that enables early mobilization and minimizes complications.

Therefore, the primary aim of this study was to evaluate the early clinical efficacy, functional outcomes, and implant survivorship of the Latitud™ total Hip Replacement System (HRS) (Meril Healthcare Pvt. Ltd., Vapi, India) in patients undergoing THA for hip fractures in the Indian clinical setting.

## Materials and methods

Study design

A post hoc analysis of a prospective, multi-center post-marketing surveillance study conducted across 11 tertiary care centers in India (CTRI No: CTRI/2017/06/008774). The study was designed to evaluate the survivorship and functional outcome of the Latitud™ HRS in patients undergoing THA. Approval for the study was granted by the Ethics Committee (Sangini Hospital Ethics Committee, India). All surgeries were performed using either the lateral or posterior approach. Patients were evaluated preoperatively and at postoperative intervals of six weeks, six months, one year, and two years.

The assessments included a thorough examination of medical history, physical examination, and radiographic imaging. Patient outcomes were measured based on predefined primary and secondary endpoints.

Endpoints

The primary endpoints of the study focused on assessing implant survivorship and the cumulative revision rate. The secondary endpoints included radiographic assessment, Harris hip score (HHS), Oxford hip score (OHS), and the documentation of adverse events throughout the postoperative to two-year follow-up period.

Eligibility criteria

Inclusion Criteria

Patients aged ≥18 years presenting with hip fractures requiring THA, including fractures of the femoral neck, femoral head, head-neck, intertrochanteric, sub-capital, and other proximal femur fractures. Patients were selected based on clinical indications for THA as recommended by the treating orthopedic surgeon, considering factors such as fracture type, bone quality, and the patient's pre-existing functional status.

Exclusion Criteria

Those patients with a known sensitivity to device materials, or those unable to provide written consent, or with a short life expectancy of >5 years (e.g., cancer, HIV) were excluded from the study.

Components of the Latitud™ hip replacement system

The Latitud™ HRS consists of several key components - (i) the Modular Shell (acetabular cup), (ii) the Modular Liner (acetabular liner), (iii) the Modular Femoral Head, and (iv) the Femoral Stem - which are available in either an uncemented or cemented form [11]. The Modular Shell is made from titanium alloy (Ti6Al4V-ELI) (ASTM F136) and is intended for cementless fixation within the prepared acetabulum. To enhance fixation, the outer surface of the shell is coated with commercially pure titanium. The Modular Liner is constructed from highly cross-linked polyethylene (HXLPE) (ASTM F648), while the Modular Femoral Head is made from cobalt-chromium alloy (ASTM F1537-1). The Femoral Stems come in two variants: the Uncemented Femoral Stem, made from titanium alloy (ASTM F136) and coated with hydroxyapatite below the resection line to improve proximal and distal fixation, and the Cemented Femoral Stem, made from high nitrogen stainless steel (ISO 5832-9), which is designed to be fixed with bone cement. Both variants feature a 12/14 taper designed to mate with the Modular Femoral Head. It is available in 11 different sizes with provision of 135° standard, 135° lateral, 125° standard (Coxa vara) neck angle, and polished distal section (Table 1).

**Table 1:** Technical specifications of the Latitud™ hip replacement system

Component	Material	Fixation	Design features	Size range
Modular Shell (Acetabular Cup)	Titanium alloy (Ti6Al4V-ELI) (ASTM F136), commercially pure titanium coating	Cementless	Outer surface coated with pure titanium	40 mm to 70 mm
Modular Liner (Acetabular Liner)	Highly cross-linked polyethylene (HXLPE) (ASTM F648)	N/A	Provides wear resistance	35 mm to 52 mm
Modular Femoral Head	Cobalt-chromium alloy (ASTM F1537-1)	N/A	Available in sizes 22 mm to 40 mm	22 mm to 40 mm
Femoral Stem (Uncemented)	Titanium alloy (ASTM F136), hydroxyapatite coating	Cementless (hydroxyapatite-coated)	12/14 taper, polished distal section, 11 sizes, 135° standard, 135° lateral, 125° standard (coxa vara) neck angle	11 sizes
Femoral Stem (Cemented)	High nitrogen stainless steel (ISO 5832-9)	Cemented	12/14 taper, polished distal section, 11 sizes, 135° standard, 135° lateral, 125° standard (coxa vara) neck angle	11 sizes

**Figure 1:**
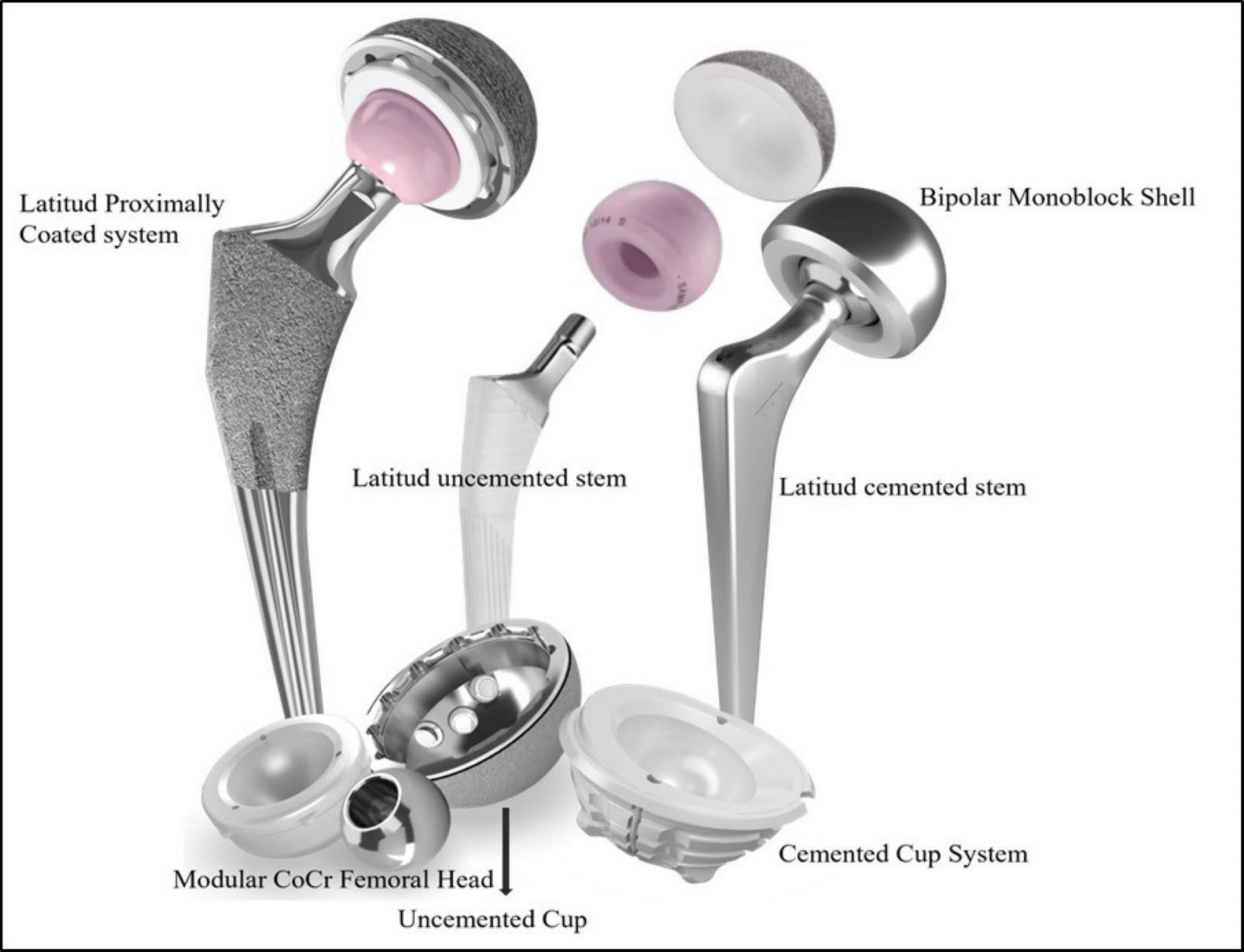
Components of the Latitud™ hip replacement system

The shell size ranges from 40 mm to 70 mm, while the liner sizes vary between 35 mm and 52 mm. The modular femoral head is available in sizes from 22 mm to 40 mm (Figure 1) [11].

The Latitud™ HRS is indicated for use in THA and hemi-hip arthroplasty. The prosthesis is aimed at improving patient mobility and reducing pain by replacing the damaged hip joint.

Data analyses

Continuous variables were represented by means along with standard deviations (SD) or range, whereas categorical variables were presented as frequencies with percentages. The comparison between preoperative and postoperative results was conducted using paired t-tests or Wilcoxon signed-rank tests, where p-values ≤ 0.05 were deemed to be statistically significant. Statistical analysis was performed using IBM SPSS Statistics (Version 28) (IBM Corp., Armonk, NY) and RStudio (Version 2022.07.1) (Posit Software, Boston, MA).

## Results

A total of 44 patients were identified with various fractures such as, femoral neck (40.9%), hip (uncategorized: 27.3%), femoral head (18.2%), femoral head-neck (4.5%), intracapsular neck of femur (ICNF) fracture (2.3%), fall at home (2.3%), sub-capital fracture (2.3%), inter-trochanteric fracture (2.3%). The average age of patients was 55.53 ± 9.83 years. The gender distribution was noted to be 28 males (63.6%) and 16 females (36.4%). Table 2 presents a comprehensive overview of the baseline demographic characteristics of enrolled patients. The patients exhibited a range of comorbidities, with hypertension (n = 3) and diabetes mellitus (n = 2) being the most common.

**Table 2:** Demographic characteristics and preoperative data of patients undergoing total hip arthroplasty with the Latitud™ total hip replacement system

Variables	Number of patients
Total patients	44
Age (years), mean ± SD	55.53 ± 9.83
Gender, n (%)	
Male	28 (62.6)
Female	16 (36.4)
Diagnosis, n (%)	
Fracture of neck femur	18 (40)
Hip fracture (uncategorized)	12 (26.7)
Fracture in femur head	8 (17.8)
Fracture of femoral head neck	2 (6.7)
ICNF fracture	1 (2.2)
Fracture due to fall at home	1 (2.2)
Subcapital fracture	1 (2.2)
Inter Trochanteric fracture	1 (2.2)
Surgical approach, n (%)	
Lateral	18 (40)
Posterior	26 (60)

**Figure 2:**
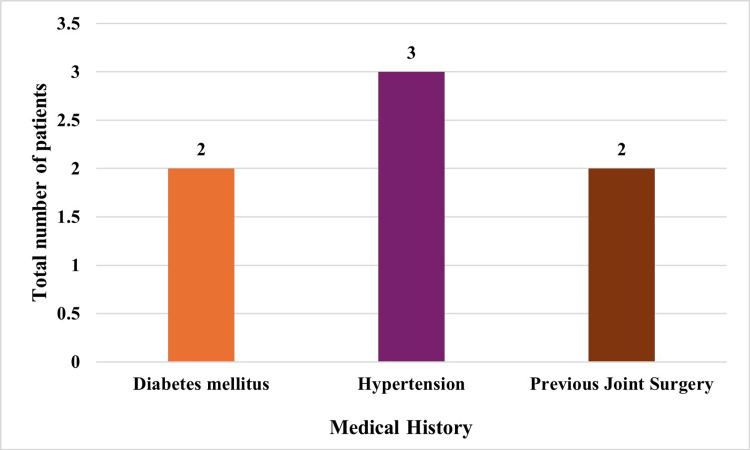
Distribution of preoperative comorbid diseases in the study population

Two patients had undergone prior joint surgeries, and one patient also had a history of pulmonary tuberculosis and bronchial asthma (Figure 2).

The surgical approach used was as follows: 18 (40.9%) of them had a lateral surgical approach, whereas in 26 patients (59.1%), a posterior approach was used (Table 2).

**Figure 3:**
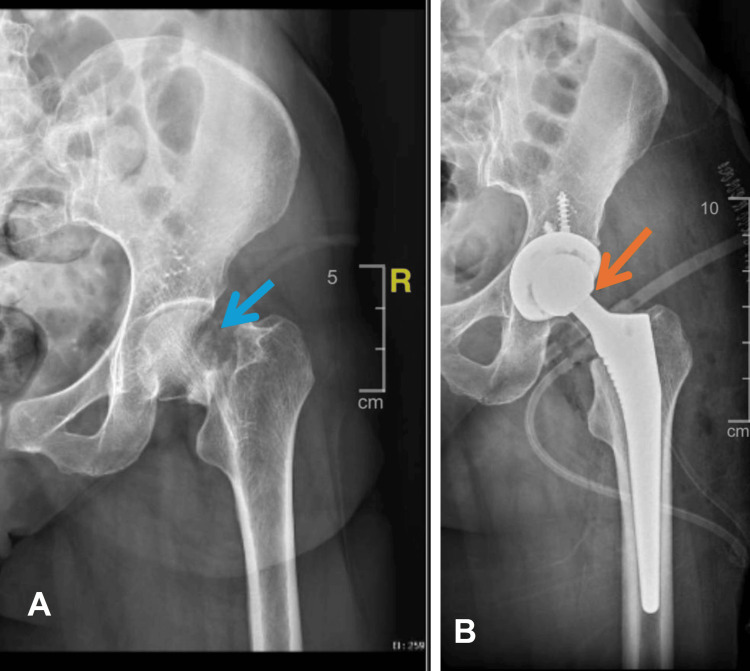
Preoperative radiograph (A) showing fracture of neck of femur (blue arrow) and postoperative (B) radiograph showing well-functional Latitud™ hip replacement system (orange arrow) implanted in a patient undergoing total hip arthroplasty

Radiographic evaluations were performed preoperatively and during follow-up assessments (Figure 3).

The study demonstrated excellent implant survivorship with the Latitud™ HRS over the two-year follow-up period. At six weeks, six months, and one year, all patients (100%) were free of reoperation, component removal, or revisions for any reason, including aseptic loosening (Table 3). By the two-year mark, implant survivorship decreased to 97.73%, reflecting one revision surgery (Table 3).

**Table 3:** Latitud™ total hip replacement system- outcomes of implant survivorship following total hip arthroplasty

Follow-up period	Implant survivorship (%)		
	Free of any reoperation	Free of component removal or revision for any reason	Free of component removal or revision of aseptic loosening
	Total number of patients, %	Total number of patients, %	Total number of patients, %
Six weeks	44 (100)	44 (100)	44 (100)
Six months	44 (100)	44 (100)	44 (100)
One year	44 (100)	44 (100)	44 (100)
Two years	43 (97.73)	43 (97.73)	44 (100)

In one patient (male, age: 59 years) with a history of diabetes mellitus, hypertension, coronary artery disease (CAD), and angioplasty, the THA procedure was performed under spinal anesthesia through a posterior surgical approach. Upon successful implantation, the patient was discharged in stable condition. During the six weeks and six months follow-up, no adverse events were reported, and the patient was clinically and functionally stable. At one-year follow-up, the patient was hospitalized with a complaint of liner breakage (squeaking or abnormal sound in left operated hip while movement) and required surgery. Revision surgery was performed after stabilizing the cardiac condition as per the cardiologist’s consultation, and the patient was performing well as per the last follow-up (Table 4).

**Table 4:** Serious adverse events and cumulative revision rate following total hip arthroplasty with the Latitud™ total hip replacement system *A 59-year-old male reported linear breakage at one year follow-up, underwent revision surgery at two years follow-up.

Events	At baseline	Six weeks	Six months	One year	Two years
Serious adverse event					
Total number of patients, n	0	0	0	1*	1
Revision rate					
Total number of patients, n	0	0	0	0	1*

In our study, the mean preoperative HHS was 9.19 ± 9.52, which consistently improved to 89.61 ± 3.57 by the two-year follow-up, indicating significant improvement in functional outcomes (p < 0.0001) (Table 5).

**Table 5:** Harris hip score following total hip arthroplasty with the Latitud™ total hip replacement system in patients with hip fractures V: Wilcox signed rank test

Scoring system	At baseline	Six weeks	Six months	One year	Two years	p-value (baseline to two years)	Test statistics (V)
Harris hip score, mean ± SD	9.19 ± 9.52	58.77 ± 12.8	73.61 ± 10.41	82.36 ± 8.19	89.61 ± 3.57	<0.0001	406

Similarly, the OHS also demonstrated marked improvement, with scores improving from 4.89 ± 4.41 preoperatively to 44.89 ± 3.77 postoperatively in two years (Table 6).

**Table 6:** Oxford hip score following total hip arthroplasty with the Latitud™ total hip replacement system in patients with hip fractures V: Wilcox signed rank test

Scoring system	At baseline	Six weeks	Six months	One year	Two years	p-value (baseline to two years)	Test statistics (V)
Oxford hip score, mean ± SD	4.89 ± 4.41	29.34 ± 8.09	36.59 ± 4.81	40.82 ± 3.42	44.89 ± 3.77	<0.0001	990

## Discussion

This post hoc assessment of hip fracture patients from the Latitud™ 180 study provides a comprehensive analysis of patients presenting with various types of hip fractures, emphasizing the demographic, clinical, and surgical outcomes over a two-year follow-up period, investigating the safety and efficacy of Latitud™ total HRS. The distribution of fracture types included fractures of the femoral neck (40.9%), hip (uncategorized: 27.3%), femoral head (18.2%), femoral head neck (4.5%) and other complex fractures such as ICNF, and intertrochanteric fractures, subcapital, neck of femur fracture due to fall at home being the most prevalent (2.3%). The patient cohort's average age was 55.53 years, and there was a male predominance (63.6%), aligning with known gender trends in trauma-associated orthopedic injuries [1]. Hypertension and diabetes mellitus were the most frequent comorbidities observed, consistent with the age-related risk factors for cardiovascular and metabolic conditions. One patient had a notable history of pulmonary tuberculosis and bronchial asthma, underscoring the varied baseline health conditions that may influence postoperative outcomes.

Femoral neck fractures, which constituted 40.9% of cases in our study, are often associated with poorer outcomes due to the challenges of maintaining stable fixation and achieving optimal healing in this anatomically complex region [12]. Published studies have reported a wide range of functional recovery rates, with some studies documenting HHS improvements from preoperative scores in the 10-20 range to postoperative scores around 80-85 at two-year follow-up [13]. In our study, however, the mean HHS improved from 9.19 ± 9.52 preoperatively to 89.61 ± 3.57 postoperatively at two years, not only falls within the upper range of reported outcomes but also demonstrates a statistically significant improvement in patient mobility and overall hip function (p < 0.0001). These results align with previous research, which similarly highlights the substantial functional gains achieved through THA [11]. In this study, the mean preoperative HHS of 42.3 ± 7.1, reflecting poor hip function, improved significantly to 91.1 ± 5.2 (range: 72-96) one year postoperatively (p < 0.001), emphasizing the procedures and device's effectiveness [11]. Comparable results were reported by Leiss et al. [14], where the preoperative HHS of 52.95 ± 12.98 improved to 91.99 ± 9.40 at 12 months, and by Karimi et al. [15], who documented a mean HHS of 99.16, further supporting our findings.

Similarly, for the OHS, improvements from a preoperative mean of 4.89 ± 4.41 to a postoperative mean of 44.89 ± 3.77 are markedly high compared to the literature. Studies have reported an increase to around 35-40 on the OHS scale, especially in older adults with comorbidities, which often limit full functional recovery [16].

Furthermore, a two-year implant survival rate of 97.7% was achieved in our cohort, which is noteworthy. Outcomes generally stabilized within the first year with implant stability, with no indication that deterioration of the observed results would occur over time [17]. The two-year implant survival rate observed in this study aligns closely with survival rates reported in established joint registries for hip fractures [18]. For instance, the National Joint Registry (NJR) documented a comparable five-year implant survival rate of approximately 97.3%, highlighting the consistency of these outcomes with broader clinical data [18].

Developing complications post-THA does not always necessitate revision surgery [19], with common causes for revision being aseptic loosening (55%), dislocation (12%), septic loosening (7.5%), and periprosthetic fractures (6%) [20]. The primary intraoperative complication encountered among THA patients is leg length discrepancy, often due to improper femoral neck length during implantation [19,21]. However, we did not observe such issues in this study.

One case of linear fracture was observed at the 12-month follow-up. This complication necessitated revision surgery. Potential risk factors for this complication include thin liner thickness, improper acetabular cup positioning, impingement, high BMI, and excessive patient activity [22]. In this study, the patient's comorbidities and elevated BMI may have contributed to the fracture, but despite the complication, the overall two-year implant survival rate remained high, underscoring the robustness of the implants in this early outcome. Comparable findings have been reported in other studies highlighting the durability of modern THA implants, even in the presence of occasional complications [11].

Interestingly, gender has been explored as a potential risk factor for THA revision, though results in the literature are mixed. One retrospective study found a higher revision rate among males compared to females [19]. No such disparity was observed in this study, and low rates of complications affirm the implant durability, safety, and efficacy of the study device.

Further, debate continues over the optimal surgical approach for THA in hip fracture patients, with the posterolateral and anterolateral approaches being the most commonly used [23]. Some evidence suggests that the posterolateral approach is associated with a higher risk of dislocation compared to the lateral or transgluteal technique [9] although other studies, such as that by Matharu et al. [24] have found superior survivorship and fewer complications with the posterolateral approach without a significant difference in dislocation-related revision risk in comparison to the anterolateral approach. In our study, both lateral and posterior surgical approaches were utilized, with the posterior approach being the more frequently employed. All patients achieved positive outcomes, and no significant adverse events were observed. However, in the single instance where revision surgery was necessitated due to a linear fracture, the posterior approach was used. While this raises questions about the possible role of surgical technique in certain complications, no definitive causal relationship was established. Overall, the lack of major complications across the cohort indicates the effectiveness of both approaches, though the case of linear fracture calls for further investigation to explore any potential link between surgical method and implant failure.

Limitations

This study has certain limitations that should be considered. The small sample size and relatively short follow-up duration may restrict the generalizability and long-term applicability of the findings. The absence of a control or comparative group limits the ability to draw definitive conclusions about the superiority of the Latitud™ total HRS. Additionally, the study relied solely on patient-reported outcomes and radiographic assessments without biomechanical evaluation. The study also did not account for potential confounders such as the variability in surgeon experience, which could influence surgical outcomes and implant performance. The multi-center nature of the study, while increasing generalizability, may also introduce inconsistencies due to differences in surgical technique and postoperative care.

Further, the absence of blinding may introduce observer bias, as the lack of blinding could influence the assessment of postoperative outcomes.

Despite these limitations, the study demonstrates several strengths. Its prospective, multi-center design across 11 Indian sites enhances the robustness and generalizability of the findings. The use of validated outcome measures, such as the HHS and OHS, ensures reliable and standardized assessment of functional outcomes, while systematic follow-up intervals allow for detailed early- and mid-term evaluations. Notably, the study reports a 97.7% implant survival rate for two years, highlighting the potential efficacy and safety of the Latitud™ total HRS. Furthermore, rigorous statistical analysis and comprehensive reporting of demographic and clinical data add to the credibility of the findings, providing valuable preliminary insights into the performance of the novel Latitud™ HRS in a real-world clinical setting.

## Conclusions

This post hoc analysis of a prospective, multi-center, post-marketing surveillance study demonstrated that the Latitud™ HRS is a reliable and effective option for managing hip fractures through THA in the Indian clinical setting. Conducted across 11 centers, this study included patients presenting with various hip fracture patterns, including femoral neck, femoral head, head-neck, intertrochanteric, sub-capital, and other proximal femur fractures. The Latitud™ HRS demonstrated excellent early survivorship with high implant survival at the two-year follow-up. Functional outcomes showed significant improvement, as assessed using validated measures including HHS and OHS. The majority of patients underwent THA using the posterior approach, while the lateral approach was also effectively utilized, demonstrating the versatility of the system in diverse clinical scenarios. Minimal complications were observed, and the single instance of revision surgery was managed effectively, indicating the durability of the implant.

In conclusion, the Latitud™ HRS has shown promising early results, indicating its potential as a reliable implant for hip fracture management in active patients. The demonstrated improvements in function and high implant survivorship underscore its utility in the Indian clinical context, supporting its adoption for THA in patients with hip fractures.
